# Longitudinal effectiveness of a woman-led, nurse delivered health promotion intervention for women who have experienced intimate partner violence: iHEAL randomized controlled trial

**DOI:** 10.1186/s12889-023-17578-4

**Published:** 2024-02-07

**Authors:** Marilyn Ford-Gilboe, Colleen Varcoe, Kelly Scott-Storey, Annette J. Browne, Susan M. Jack, Kim Jackson, Tara Mantler, Sue O’Donnell, Noël Patten-Lu, Victoria Smye, C. Nadine Wathen, Nancy Perrin

**Affiliations:** 1 Arthur Labatt Family School of Nursing, Western University, 1151 Richmond St, London, ON NBA 5C1 Canada; 2 School of Nursing, University of British Columbia, Vancouver, BC Canada; 3 Faculty of Nursing, University of New Brunswick, Fredericton, NB Canada; 4 School of Nursing, McMaster University, Hamilton, ON Canada; 5 School of Health Studies, Western University, London, ON Canada; 6 School of Nursing, Johns Hopkins University, Baltimore, MD USA

**Keywords:** Intimate partner violence, Domestic violence, Randomized controlled trial, Complex interventions, Health outcomes, Quality of life, Post-traumatic stress disorder, Depression, Chronic pain, Nursing

## Abstract

**Background:**

Intimate partner violence (IPV) threatens the safety, health and quality of life of women worldwide. Comprehensive IPV interventions that are tailored, take a long-term view of women’s needs, including health concerns, and maximize choice and control, have the potential to effectively address heath and safety concerns. Few such interventions have been tested, including in the Canadian context.

**Methods:**

A parallel randomized controlled trial of adult (age 19 + years), English-speaking, Canadian women with histories of IPV randomized either to iHEAL, a tailored health promotion intervention delivered by Registered Nurses over 6–7 months, or to community service information (usual care control). Primary (Quality of Life, PTSD symptoms) and secondary outcomes (Depression, Confidence in Managing Daily Life, Chronic Pain, IPV Severity) were measured at baseline and 6, 12 and 18 months post-intervention via an online survey. Generalized estimating equations were used to test for differences by study arm in intention-to-treat (full sample) and per protocol (1 + iHEAL visit) analyses focussing on short-term (immediately post-intervention) and longer-term (1 year post-intervention) effects. Selected process evaluation data were summarized using descriptive statistics.

**Results:**

Of 331 women enrolled, 175 were randomized to iHEAL (135 who engaged in 1 + visits) and 156 to control. Women who received iHEAL showed significantly greater short-term improvement in Quality of Life compared to the control group, with these effects maintained 1 year later. Changes in PTSD Symptoms also differed significantly by group, with weaker initial effects that were stronger 1 year post-intervention. Significant moderate, short- and longer-term group effects were also observed for Depression and Confidence in Managing Daily Life. IPV Severity decreased for both groups, with significant immediate effects in favour of the intervention group that grew stronger 1 year post-intervention. There were no changes in Chronic Pain.

**Conclusion:**

iHEAL is an effective, acceptable and safe intervention for diverse groups of women with histories of IPV. Trial results provide a foundation for implementation and ongoing evaluation in health care settings and systems. Delayed effects noted for PTSD Symptoms and IPV Severity suggest that longer-term assessment of these outcomes may be needed in trials of IPV interventions.

**Trial registration:**

Clinicaltrials.gov ID NCT03573778 (Registered on June 29, 2018).

## Background

Intimate partner violence (IPV) is a complex, global health and human rights issue and a leading cause of death, disability and illness among women [1, 2]. In Canada, more than 4 out of 10 women who have ever been in an intimate relationship report having experienced IPV [3]. There is substantial evidence documenting the significant and *enduring* toll of IPV on women’s safety and security, relationships, economic situations, quality of life and mental and physical health, both for women living with an abusive partner and after separation [4–6].

Attention to the *safety* of women who have experienced IPV is critical, yet women’s needs extend beyond safety. In the context of separation, for example, women’s priorities often shift to include: accessing adequate housing and creating economic stability [7]; supporting children and dealing with custody and access disputes [8, 9]; managing chronic health concerns [10]; and accessing help from complex systems that are confusing and, at times, unresponsive [10, 11]. The impact of these issues, and the challenges of dealing with them, are magnified for women who face structural inequities of poverty, stigma and/or discrimination, including Indigenous women [12, 13], newcomers [14], and those living in underserved communities, including in rural settings [15]. In the context of chronic stress, women’s efforts to manage their lives in ways that support their recovery, safety, health and well-being may be undermined, at considerable cost to women, families, communities and society [16].

Although women who have experienced IPV access health services at higher rates than women in the population [17], most services have not been designed to take women’s experiences of violence into account. Indeed, women often report negative and dismissive experiences, with research specifically documenting the need to improve healthcare responses to IPV [18, 19]. Until recently, existing guidelines for health professionals have focussed on routine screening for IPV. While screening can identify women experiencing IPV, there is no evidence of its effectiveness in improving women’s health or reducing violence, in the absence of a follow-up intervention [20, 21]. Attention has shifted toward improving the capacity of providers and organizations to provide safe, trauma- and violence-informed services for all [22], and offering more specialized, evidence-based interventions to women when IPV has been identified [20], particularly interventions that are trauma-and violence-informed [23].

There is growing trial evidence supporting the *short-term* effectiveness of health and safety interventions in improving the mental health of women who have experienced IPV [24–27]. Results of systematic reviews suggest that specific types of IPV interventions are effective in improving some outcomes in some contexts. For example, a recent Cochrane Review [24] showed that psychological therapies focussed on reducing distress and promoting healing from trauma probably reduce depression and possibly reduce anxiety (6–12 months after therapy), but do not appear to affect other outcomes, including post-traumatic stress disorder (PTSD), quality of life, and exposure to violence. Results of a realist review [26] support varied effects of advocacy interventions (i.e., those providing legal, housing or financial advice or education, access to safety planning, support and system navigation); specifically, more intensive (12 + hours) but not less intensive advocacy appears to improve women’s quality of life and reduce physical abuse (1–2 years post-intervention) for women leaving a shelter, but there is limited quality evidence that advocacy benefits women’s mental or physical health. No Canadian studies were included in these reviews. Further, there is limited evidence that IPV interventions offered in health care settings positively affect women’s safety, or their physical health [24, 25]. These gaps are important since violence leads to negative social, economic and health outcomes [4–6], including chronic physical health challenges, a main contributor to the global burden of disease among women [28].

Further, there is a mismatch between IPV interventions that take a short-term perspective and focus on specific issues (such as safety, system navigation or mental health) and conceptualizations of IPV as a complex trauma that persists over time and has broad impacts [29]. Few trials have examined longer-term effectiveness of complex IPV interventions, including for women in the transition of separating from an abusive partner. More holistic, theoretically grounded, trauma- and violence-informed interventions tailored to fit with women’s needs and lives are needed, particularly those that concurrently address the health consequences of IPV, women’s safety, and the conditions that create barriers to improvements in health and well-being. Indeed, a recent synthesis of qualitative studies [30] supports that women experiencing IPV desire respectful, consistent support that addresses the full spectrum of their needs and concerns, including complex health issues and access to community resources. To advance existing knowledge, research testing such interventions should use rigorous designs that incorporate attention to processes of change and consider longer-term (sustained) effects of intervention on a range of outcomes.

### Intervention for health enhancement and living (iHEAL)

To address existing gaps, we developed the Intervention for Health Enhancement and Living (iHEAL), an evidence-based, health promotion intervention designed to support women in the transition of separating from an abusive partner to identify and manage health and other concerns [31]. iHEAL is informed by the qualitative grounded theory *Strengthening Capacity to Limit Intrusion* [10] which described the multiple priorities of women who had separated from an abusive partner and the concurrent ‘intrusive’ challenges they faced as they worked to create a different life for themselves and their children. This theory gave rise to the six components of the intervention, each of which focusses on an issue known to affect women’s well-being. The breadth of iHEAL, where nurses focus concurrently on women’s physical and emotional safety, health and well-being, relationships and connections with others, and basic needs over time as they negotiate the transition of separation, and it’s trauma-and violence-informed, equity-oriented approach, is novel among IPV interventions. iHEAL addresses an important gap in interventions for women experiencing IPV by taking a long-term perspective on women’s needs for support, since many interventions and services focus on the crisis period around leaving, and fewer on the longer-term issues and needs of women across the process of separation (including while trying to create a life separate from the partner). Based on it’s theoretical grounding and research base, we designed iHEAL to be appropriate for women who have named their relationship as abusive and are taking steps to address this in some way. This does not have to be by separating, although the majority of women who experience IPV in the Canadian context do eventually separate from their abusive partners.

iHEAL nurses are Registered Nurses (RNs) with a minimum of a baccalaureate degree who complete an intensive educational program to prepare them to deliver the intervention. RNs in Canada have a scope of practice that encompasses the areas of intervention addressed by the 6 iHEAL components. While there is overlap with other disciplines (e.g. managing symptoms overlaps with primary care provision), nurses bring a unique perspective and skill set to each component (e.g. non-pharmacological pain management), especially related to the health effects of violence and related interventions, the health of the self, basic needs that extend beyond material resources (e.g. having energy for daily life in the face of depression, trauma symptoms, or chronic pain), and family dynamics, a crucial consideration in the context of IPV. Nursing practice in iHEAL is guided by Relational Inquiry [32], a nursing practice approach based on complexity theory that directs practice to focus simultaneously on: (a) the individual’s inner life (in this case, women’s priorities, agency, confidence, etc. and nurses’ potential bias, lived experiences, etc.), (b) engagement between and among individuals (in this case between the woman and nurse, between the women and their ‘others’, including other service providers, etc.), and, (c) the context (e.g. availability of resources, dominant understandings of and social responses to violence against women, etc.).

The nurses’ education sessions focus on the application of iHEAL principles and components, as well as delivery, safety and documentation protocols. Using interactive activities, assignments, and small group discussion, the sessions prepare nurses to offer iHEAL with fidelity to the intervention principles and components. Building on the broad education of RNs, we provide additional education in areas that include the dynamics of IPV, substance use health, pain management and cultural safety. Critically, taking a woman-led, non-judgemental approach, the education emphasizes the importance of supporting women’s priorities and decisions, including the decision to remain with the partner, an aspect of IHEAL that women routinely express as important and impactful [33, 34, 36].

**Fig. 1:**
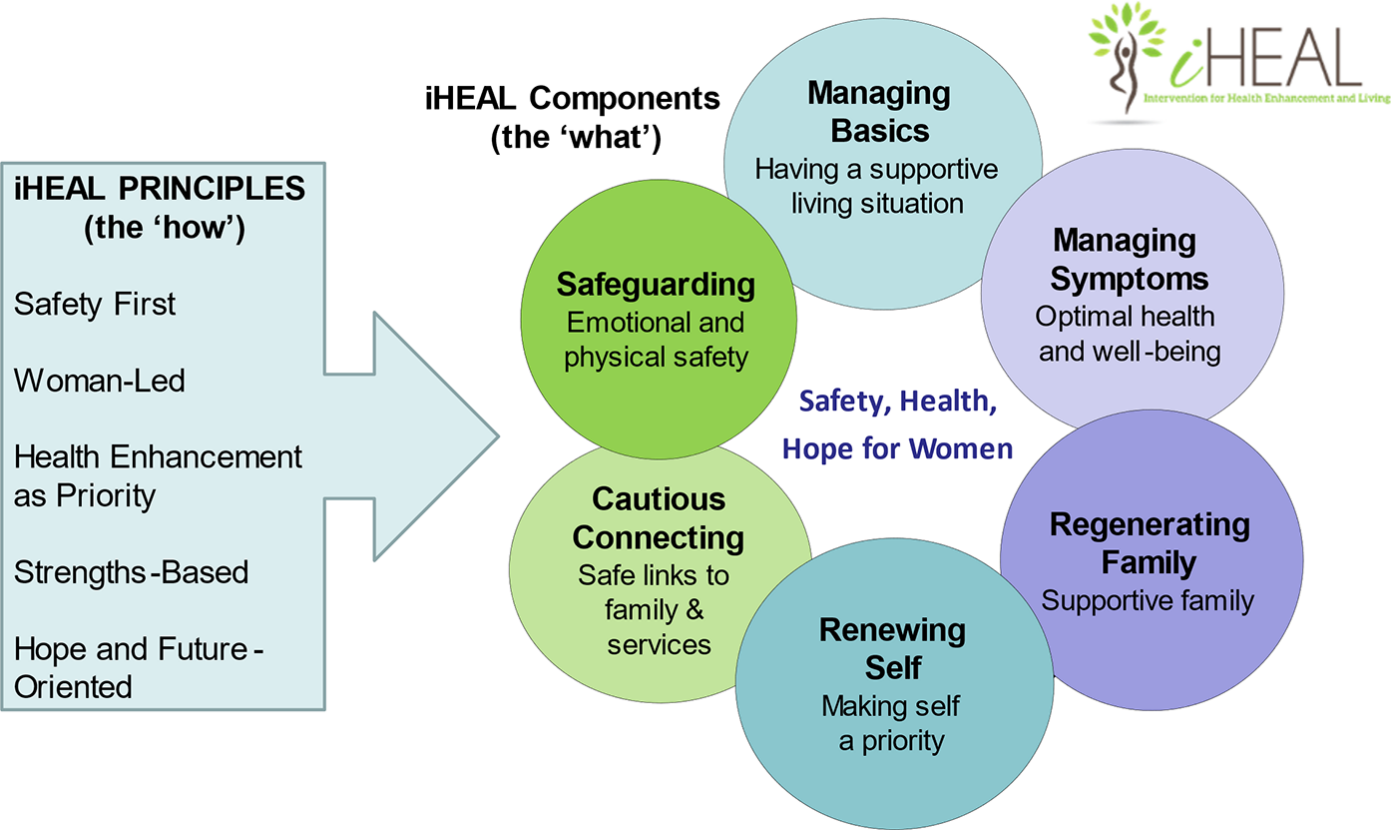
The principles and components of iHEAL

iHEAL nurses work with women over a 6–7 month period using a flexible, three-phase process of tailored support to help the woman identify and address both her short-and longer-term priorities and goals for health, well-being, and safety [31]. In iHEAL, the focus of the nurse-woman encounters is on developing the woman’s capacity to manage the multiple issues she faces over the long-term in ways that build her knowledge, skills, confidence, and resources and complement and extend existing services. Drawing on the five iHEAL principles, nurses help each woman consider her experiences, strengths, needs and priorities in relation to the six intervention components (Fig. 1). The three-phase process is tailored to each woman’s priorities, preferences and living situation and to the local community context, requiring that nurses get and stay ‘in sync’ with the woman. A summary of key activities in each phase is provided in Table 1. Additional details can be found elsewhere [31, 34].

In the first 2–3 visits (*Getting in Sync*), the nurse explains the intervention, clarifies expectations, and explores the woman’s priorities. Over the next 4–5 months (*Working Together*), the nurse introduces each component and explores how it is relevant to the individual woman. The woman directs the order and how much time is spent on each intervention component. In this phase, the nurse provides information and support to help the woman assess her current strengths and develop her own knowledge, skills and resources to manage the issues affecting her health, safety and wellbeing. For example, for women struggling with chronic pain, nurses assess the severity and impact of pain, discuss options for managing the pain (such as acupuncture, mindfulness, or connecting her to specialized pain management services) and/or work with women to get support from family members, friends or services. In offering iHEAL, nurses draw on knowledge and skills developed through standardized iHEAL education and the *iHEAL Practice Guide for Nurses*, complemented by their existing expertise as Registered Nurses.

In the final 3–4 weeks (*Moving On*), iHEAL nurses help women plan to continue caring for their health and well-being after iHEAL has ended. This includes supporting each woman to reflect on her experiences of iHEAL, offering a letter outlining her participation (a record for themselves or to share with others if they choose) and continuing to help her access supports and services that could provide longer-term assistance as needed. To celebrate the woman’s engagement in iHEAL, the nurse provides the woman with a certificate of completion and a small gift (e.g., a journal).

**Table 1:** Key iHEAL intervention activities across a three-phase model of delivery

iHEAL Phase	Focus and Key Activities
**Getting in Sync** (first 2–3 visits, then ongoing to maintain)	**Setting a foundation for relational engagement** • Explain the intervention • Discuss the woman’s expectations • Negotiate visits • Explore the woman’s priorities • Listen to her “Story of Survival”
**Working Together** (4–5 months)	**Providing tailored information and support to assist the woman to address key needs** • Explore the relevance of each component to the woman’s experiences, needs and future goals • Identify options for addressing each priority • Complete 2 required tools: Danger Assessment [44] and Symptom Checklist • Offer tailored supports as negotiated with the woman
**Moving On** (last 3–4 weeks, overlapping with working together)	**Planning to Sustain the woman post-intervention** • Engage the women to reflect on progress, needs, goals • Identify where ongoing support/connection would be helpful • Support women to access services and supports as needed and preferred • Celebrate successes (certificate, small gift)

iHEAL has been tested in 3 feasibility studies with two general community samples of women in Ontario (N = 29) and New Brunswick (N = 56) [34], and with Indigenous women in British Columbia (N = 152) [35, 36]. Each study employed a single group, before-after study design with data on the same core outcomes collected at 3 time points: baseline (pre-intervention), 6 months (post-intervention) and 12 months (6 months after the intervention ended), along with a process evaluation exploring feasibility, acceptability, unanticipated impacts and areas needing refinement. All studies supported iHEAL’s acceptability and feasibility of delivery at reasonable cost (~$2600 CAD per woman). Further, women who took part in iHEAL showed significant post-intervention improvements in key areas, including quality of life, mental health, control and confidence in managing daily life, chronic pain (Ontario only) with these changes maintained 6 months after the intervention ended. While these results are promising, without a control group, it is unclear whether positive changes observed in these studies can be attributed to the intervention or to the passing of time or women’s own help seeking. To address this gap, in this study, we conducted a randomized controlled trial (RCT) of iHEAL tested against usual care, with a focus on both immediate and longer-term effectiveness.

### Objectives of the trial

The primary aim of this study was to test the effectiveness of iHEAL in improving quality of life, health, confidence, and safety outcomes. Women randomized to iHEAL nurse visits were compared to a usual care control group. We hypothesized that, when compared to usual care, iHEAL would improve women’s Quality of Life and reduce PTSD Symptoms (primary outcomes), Depression and Chronic Pain, increase Confidence in Managing Daily Life, and reduce Severity of IPV (secondary outcomes) immediately post intervention (6 months), with these effects maintained 1 year post-intervention (18 months).

Consistent with recommendations for testing complex interventions [37], this trial incorporated a process evaluation to explore the mechanisms underlying intervention effects (if any) and women’s experiences of iHEAL along with analyses to examine fidelity, costs, and differential effects of the intervention for specific groups of women. A related sub-study explored nurse’s experiences of iHEAL education and delivery on their practice. The results of these analyses are not reported here but are important for contextualizing the effectiveness results, with implications for broader implementation and scale up.

## Methods

### Trial design

Between September 26, 2018 and May 15, 2021, we conducted a parallel, RCT to test the effectiveness of iHEAL among Canadian women living in three provinces (BC, Ontario, New Brunswick). Women were randomly assigned to receive iHEAL nurse visits over approximately 6–7 months, or brief information about local services to use on their own as a proxy for usual care (control group). The provision of information about local resources and services is consistent with recommendations for first-line support of women who disclose IPV to health care providers [21]. Women completed online surveys comprised of outcome measures at baseline and 6, 12, and 18 months later, and those in the intervention group completed additional questions assessing acceptability, safety, harms and fidelity of iHEAL immediately post-intervention (6 months). After the 18 month-survey, a subsample of 29 women also completed a qualitative interview about their experiences.

The study protocol was developed using the CONSORT Statement for RCTs [38] and approved by the institutional Research Ethics Boards at the University of Western Ontario, University of British Columbia and University of New Brunswick in July, 2018. A Data Safety Monitoring Committee (DSMC) met four times during the trial to review the use of study protocols and any issues related to safety, with no adverse events or concerns identified; study continuation without change was recommended following each meeting. Pandemic restrictions due to COVID-19 required that nurse visits for the last 2 women in the intervention be completed by phone or online. While baseline surveys were completed prior to the onset of the pandemic, many follow up surveys (post-interventon, 6 and 12 months later) were conducted when physical distancing restrictions were in place. However, because surveys were completed online, they were minimally disrupted. Qualitative interviews, which began after all women had completed the 18 month survey, were all conducted by phone or virtually.

### Participant recruitment and enrollment

Participation in this study was open to English-speaking women (inclusive of Trans women) aged 19 years or older who reported experiencing physical, sexual and/or emotional abuse by a current or former partner of any gender in the previous 12 months (based on a modified version of the Abuse Assessment Screen [39]; were in the transition of separating from that partner (defined as planning or taking steps to separate, or having separated from an abusive partner in the previous 3 years); lived in one of the study sites (in the provinces of British Columbia, Ontario, or New Brunswick) and planned to stay in the area for at least 6 months. Women were also required to have a safe phone number and email address to enable enrollment, communication and intervention delivery, and access to a device (computer, table or phone) with internet to complete surveys. Those who did not meet all criteria were excluded from participation.

Participants were recruited primarily using online advertisements (e.g., classified ads, websites of services or groups; social media) supplemented by flyers posted in community settings accessed by women (e.g., libraries, community centers). Women were directed to a secure study website to obtain study information, complete eligibility screening and receive an immediate response about their eligibility based on an algorithm programmed into the website. Those who were ineligible were thanked and provided with contact information for their provincial domestic violence helpline. Eligible women were asked to read the study Letter of Information and, if interested, provide written consent online and enter information (i.e., name, address, safe email address, phone numbers, specific instructions for contact) into the website to enable the research team to safely contact them throughout the study.

Given that interactions between highly skilled research staff and participants have been suspected of diluting intervention effects in other IPV trials [40, 41], we opted for completely online enrollment and data collection. However, in the first 2 weeks of the trial, we encountered challenges associated with online recruitment including missing contact information, and attempts to enroll by women living outside the catchment areas, and/or who did not understand the nature of the study (e.g., possible assignment to either arm; intervention lasting approximately 6 months). To promote fully informed consent and enrollment of eligible participants, after women completed online eligibility screening and initial wrttten consent, we added a brief phone contact with a Research Assistant to validate the woman’s eligibility and safe contact information, answer questions about the study and re-affirm consent. If a validation phone call could not be completed after several attempts, women were not enrolled in the study. Enrollment was completed 12 months after study initiation (in August 2019).

### Power calculation and sample size

The statistical power analysis used means and standard deviations from iHEAL pilot study data from Ontario and British Columbia with Quality of Life (M = 37.69, SD = 10.17, N = 64) and PTSD (M = 59.61, SD = 26.98, N = 64) as the primary outcomes. The statistical model is based on repeated measures analysis of variance rather than the planned Generalized Estimating Equations (GEE) approach because the specific estimates of variance needed under the GEE model were not available. The parameter of interest is the group by time interaction. We planned to recruit 280 women (140 per group) based on the ability to detect group by time effects of 0.34 or greater (i.e., moderate effects) with power = 0.80 and alpha = 0.05. No adjustment was planned based on attrition since the GEE model does require complete data from each participant at all time points [42].

### Trial procedures

As women were enrolled, a secure online tracking data base automatically generated a study ID and sent an email message inviting the woman to complete the baseline survey using a unique URL. Women were advised that surveys could be completed in more than one session if needed, with responses saved. Automated and manual reminders from Research Assistants were sent by email up to 4 times to encourage survey completion. Those who completed the baseline survey within 14 days accrued to the trial and were sent links to follow-up surveys at 6, 12 and 18 months, with regular reminders until those surveys closed after 28 days. Those who did not complete the baseline survey within the enrollment period were sent an email stating that they had not been enrolled and would receive no further study communication.

Surveys were comprised of outcome measures and demographic questions, and took 30–45 min to complete. At the end of each survey, women were invited to leave comments about their experiences and were provided with information about the signs of a stress response and strategies to manage distress as part of a safety protocol [40], as well as an honorarium of $30 CAD as an electronic giftcard sent to the woman’s safe email address. At 6 months, the intervention group survey included questions evaluating acceptability, safety, harms and fidelity of iHEAL delivery. At 18 months, all women were asked about to indicate their interest in completing a qualitative interview about their study experiences.

### Randomization

Submission of the baseline survey activated computer randomization of participants to a study arm (iHEAL nurse visits or usual care) at a 1:1 ratio using a minimization scheme that considered province of residence. The randomization algorithm was pre-programmed into the study tracking database by the programmer who had no contact with participants. Email messages were sent from the tracking data base to the woman (to advise her of the group assignment) and to the Clinical Supervisor (to enable assigning a nurse to work with the woman). Since the use of automated online data collection and reminders minimized contact between research staff and participants, staff were not blinded to group assignment.

### Intervention arm (iHEAL nurse visits)

#### Preparation for the trial

Using insights from our previous studies, we made several changes to enhance the intervention including: (a) consolidating iHEAL principles that operationalize the theoretical base of iHEAL and guide ‘how’ to work with women from the original 10 to 5; (b) adopting Relational Inquiry as an approach to nursing practice [32], and (c) fully integrating the concepts of equity-oriented care, trauma- and violence-informed care (TVIC), cultural safety and harm reduction to deepen attention to structural conditions shaping women’s experiences. Because Indigenous women experience significantly more violent victimization, including IPV, than other Canadian women [43], we committed to supporting nurses to tailor the intervention to the specific needs of Indigenous women in each site. These changes reinforced the importance of nurses adopting a stance of humility to prioritize each woman’s knowledge, experience, emotional and cultural safety, and choice and control, while also encouraging nurses to advocate for changes to address systems issues that negatively affect women’s lives.

We also developed new resources to support intervention delivery that included: (a) 8 *online learning modules* that introduce the concepts, principles and structure of iHEAL and orient the learner to key topics (e.g., IPV and its’ health and social impacts, women’s experiences in the transition of separation, the use of TVIC and relational inquiry, cultural safety and harm reduction); (b) a more refined *iHEAL curriculum* and plan for face-to-face learning; (c) the *iHEAL Practice Guide for Nurses*, a 200 + page manual desiged to support intervention delivery, with guidelines, sample activities, and practical examples; (d) a *Woman’s Workbook*, with information and activities presented in a user-friendly format; and (e) a *Clinical Supervisor’s Guide*, which included suggestions for clinical, administrative and reflective supervision of iHEAL nurses. Clinical Supervisors are senior Registered Nurses with a minimum of 3 years administrative/management experience, who provided direct support and mentoring for nurses, in addition to offering iHEAL to a small caseload of women.

#### Education of nurses and clinical supervisors

The nurses received 58 h of initial education. Phase 1 (iHEAL Introduction) involved completing approximately 26 h of online learning comprised of: (a) the 8 iHEAL online modules (approximately 14 h); (b) a two-hour certification in the use of the Danger Assessment, a standard risk assessment for serious or lethal IPV [44]; and (c) a 10 h Indigenous Cultural Safety training program called San’yas [45]. Phase 2 (iHEAL Fundamentals) consisted of 32 h of face-to-face, group-based, applied education focussed on knowledge and skills development to offer iHEAL with fidelity. Sessions were facilitated by members of the research team who developed iHEAL, all of whom are experienced educators. Clinical Supervisors completed 2 additional days of training to prepare them for their supervisory roles.

#### Intervention delivery, tracking and documentation

We created a custom-built, password protected, online system to track women’s progress through the trial that included functions to assign nurses to work with individual women, to safely contact participants, and to document and track each woman’s participation in the iHEAL visits. Clinical Supervisors received an email notification when a woman was assigned to the intervention arm and assigned a nurse to work with the woman based on caseload. Nurses were then notified of the assignment and used the woman’s safe contact information and safety protocols to arrange a first visit. Protocols for setting up visits took into account the reality that separation is often a long-term, fluid process of leaving and returning, emphasizing the safety of women and nurses at all times; the expectation was that women’s situations would change, and that nurses would not expect women to separate, but support their decisions, focusing on their well-being. Thus, nurses continually negotiated the context of each visit. All initial visits, and subsequent visits with women who lived with an abusive partner, took place in a safe, private community location, normally in a public space, such as a library or service setting. For women not living with an abusive partner, the location of visits was negotiated based on safety, privacy, practical barriers and women’s preferences. For all women, nurses made visits every 1–2 weeks for a total of 10–18 visits over approximately 6 months, but with flexibility to fit with the woman’s needs.

Standard post-visit documentation included the timing and type of visit (in-person, phone, text), issues discussed, activities completed, outcomes, referrals made and services used by the woman and, with the woman’s permission, uploaded copies of completed intervention activities (e.g., self-assessment tools). Completion of the intervention was noted by nurses and verified by supervisors; this activated the automatic deployment of the post-intervention (6 month) online survey.

#### Clinical supervision and team support

Ongoing support and mentoring of nurses was provided by the Clinical Supervisor who facilitated local team meetings every 2 weeks to engage in case review and problem-solving, information sharing and debriefing as a group. The Clinical Supervisor also provided 1:1 reflective supervision with each nurse on a monthly basis. Clinical Supervisors met with the research leads (at their site and across sites) weekly or biweekly to problem-solve any implementation issues and ensure fidelity within and across sites. Clinical Supervisors also met virtually as a group every 4–6 weeks to share best practices and to support each other in this role.

### Control arm (usual care arm)

Women who were randomized to the control group received a link to a PDF document containing basic information about how to access domestic violence and other crisis services in their province. The email message stated that women were free to use this information as they wished.

### Outcomes

Two primary and four secondary outcomes were assessed using self-report measures that have demonstrated reliability and validity in previous samples from the study population. Higher scores on each measure reflect higher levels of the specific outcome.

#### Primary outcomes

*Quality of life* was measured using the Quality of Life Scale (QOL) [46], a 9-item unidimensional, self-report scale specifically designed to capture global satisfaction with life across nine domains (e.g. life as a whole, oneself, family responsibilities, personal safety, independence and freedom). It was developed for use with women who have experienced IPV and used as the primary outcome in IPV intervention studies [46, 47], including in iHEAL feasibility studies [34, 36]. The scale has demonstrated reliability, responsiveness to change, and structural validity [48]. Items are rated on a 7-point Likert scale ranging from ‘extremely pleased’ (1) to ‘terrible’ (7), and are reverse scored and summed to produce total scores ranging from 9 to 63. Internal consistency (Cronbach’s alpha) in this study was 0.91.

*PTSD Symptoms* were measured using the 17-item PTSD checklist, Civilian Version (PCL-C), a self-report measure designed for use in community samples to assess the probability of meeting DSM-IV diagnostic criteria for PTSD [49, 50]. The PCL-C asks respondents to rate how bothered they have been by symptoms linked to stressful experiences in the previous month, using a 5-point Likert-type scale, with responses from 1 (not at all) to 5 (extremely). Total summed scores range from 17 to 85, with higher scores indicative of greater symptomatology. In this study, internal consistency (Cronbach’s alpa) was 0.92.

#### Secondary outcomes

*Depression* was measured using the 20-item Center for Epidemiologic Studies Depression Scale, Revised (CESD-R) [51, 52], a widely used self-report scale shown to be reliable and valid in varied populations, including in prior iHEAL feasibility studies [34, 36]. Women rated how often, in the previous week, they had experienced each symptom using a 4-point response scale that ranged from ‘not at all or less than 1 day’ (0) to ‘nearly every day for two weeks’ (3). Total scores, ranging from 0–60, were computed by summing responses to applicable items. Internal consistency of the CESD-R in this study was .94.

*Chronic Pain* was measured using the disability score from Von Korff’s Chronic Pain Grade [53] to capture the impact of chronic pain on an individual’s life and functioning. Scores range from 0 to 10 and are computed as the mean score of 3 items where women were asked to rate the degree to which pain interfered with usual activities on a 10 point scale from ‘does not interfere’ (0) to ‘completely interferes’ (10). In this study, internal consistency was .93.

*Confidence in Managing Daily Life* was measured using a 10-item scale developed for this study using Bandura’s [54] methodology. Women were asked to rate their confidence in engaging in actions that reflect the 6 iHEAL components on a visual analogue scale (VAS) ranging from ‘not at all confident’ (0) to ‘completely confident’ (100). Sample items include: *make a plan for keeping yourself safe*, *manage your health problems*, *get the services you need, make time for yourself.* VAS scores were recorded by the website as the distance in millimetres from the left anchor (0) to the location of the mark on the line. Total scores, computed as the mean of item scores, range from 0 to 100, with higher scores representing greater confidence. Internal consistency across the 10 items was 0.86.

*Severity of Intimate Partner Violence* was measured using the Composite Abuse Scale (Revised)-Short Form (CAS_R_-SF) [55], a 16-item summated rating scale developed to capture severity of physical, sexual and psychogical abuse, including coercive control, from a current or former intimate partner. Respondents rate how often they have experienced each item in the previous 12 months on a 6 point-likert scale with options ranging from 0 (never) to 5 (daily or almost daily). Total scores, created by summing responses, range from 0 to 80. Internal consistency in this study was 0.91.

### Statistical analysis

Chi-square and t-tests were used to determine if randomization achieved balance between the two groups at baseline. Generalized Estimating Equations (GEE) with a Gaussian distribution were used to examine the difference in change over time between the intervention and control groups for the primary outcomes of Quality of Life and PTSD Symptoms as well as the secondary outcomes (Depression, Chronic Pain, Confidence in Managing Daily Life, and Severity of IPV). Time (baseline, 6, 12, and 18-months), group (intervention or control) and the time by group interaction were included in the model with a focus on short-term (immediately post-intervention) and longer-term (1 year post-intervention) effects. GEE allows for missing data across time so all participants were included in the analyses [42]. Both Intention-to-Treat (ITT) and Per Protocol (PP) analyses were conducted. Effect sizes were estimated using Cohen’s d.

## Results

### Recruitment, randomization and retention

**Fig. 2:**
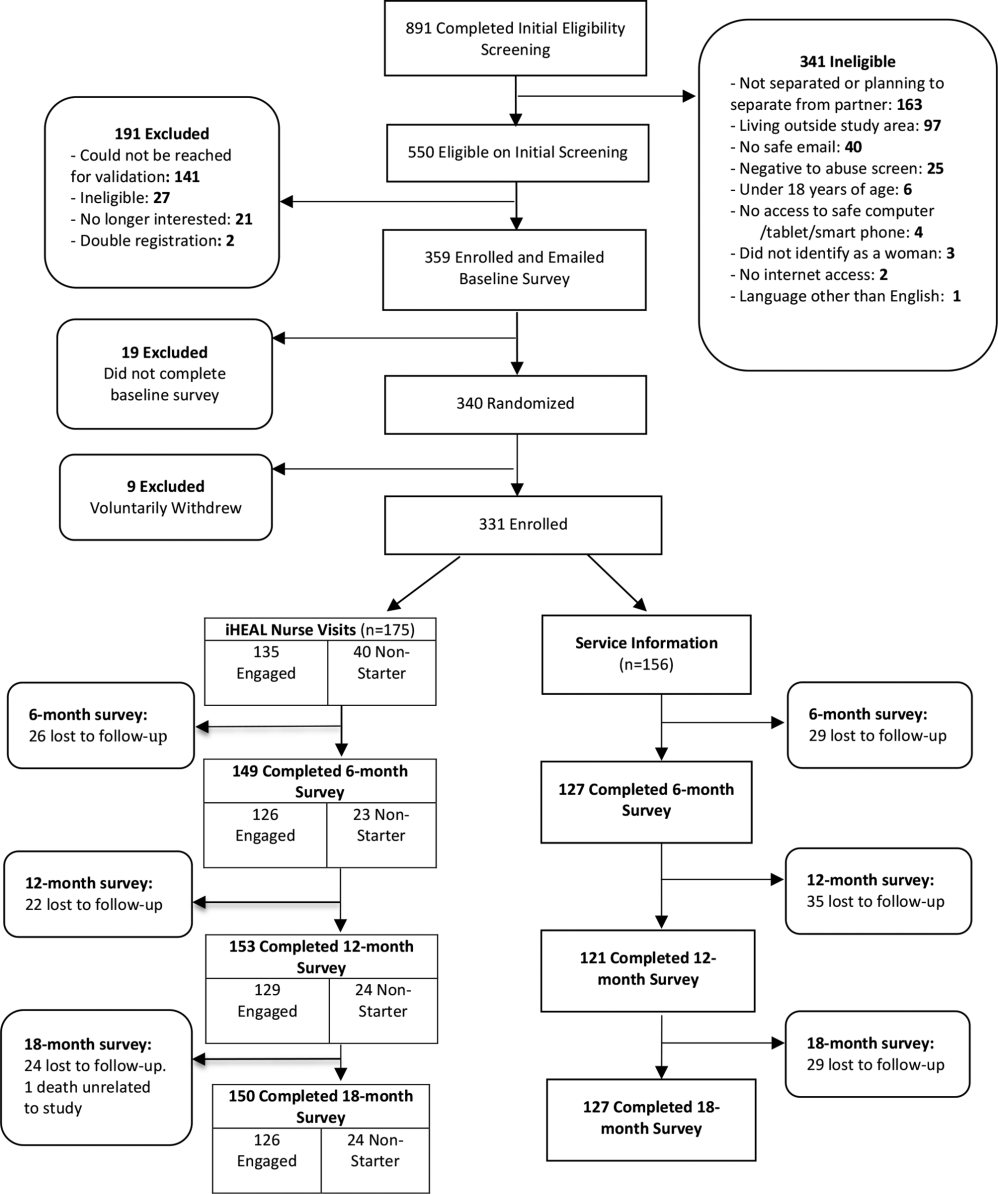
Consort diagram

Of the 891 women who completed initial online eligibility screening, 341 were deemed ineligible for a variety of reasons, most commonly because they had not separated/were not planning to separate from an abusive partner (n = 163) or lived outside the study catchment area (n = 97) (Fig. 2). Of the 550 women who passed the initial online screening, 191 were excluded as they could not be reached to validate their enrollment (n = 141), were ineligible (n = 27), no longer interested (n = 21) or enrolled twice (n = 2). A total of 359 women were enrolled and emailed a link to the baseline survey. Overall, 340 of these women completed the baseline survey and were randomized; however, 9 women withdrew from the trial after randomization but before intervention because they were unable to commit to nurse visits (due to time, chaos in their lives or moving out of the area). Thus, 331 women remained (175 in the intervention group, 156 control group). Overall retention was 83.4% at 6 months, 82.8% at 12 months and 83.7% at 18 months.

Of the 175 women randomized to the intervention, 135 women (77%) engaged in at least one visit with the nurse, while 40 did not start the intervention (i.e., no visits made). Reasons for not starting the intervention included that the nurse was unable to contact the woman (14) or made contact but could not set up a visit (22), safety concerns with partner (2), receiving supports elsewhere (1) or moved out of the area (1). To mitigate the impacts of non-engagement in the intervention, and ensure that groups of comparable size would be available for the per protocol analysis, 6 months into the trial we shifted the randomization from 1:1 allocation to 3:2 in favour of the intervention group. This change was made on May 9, 2019 after reviewing the enrollment data and identifying the issue with non-engagement.

### Sample demographics

The average age of participants was 35.5 years (range 19 to 69 years) (See Table 2). A little less than half (43.8%) were parenting a child or children under 18 years. The intervention group participants were slightly older (36.8 vs. 34.1 years) and were more likely to have children under 18 years (49.1% vs. 37.8%). There were no other demographic differences between groups. The majority (43.5%) of women had graduated from college or university. Half (50.5%) were unemployed, with 45.0% reporting that it was difficult or extremely difficult to live on their current income. Women lived in communities that included rural areas/small towns (11%), medium-size cities (31.4%) and large cities (58%). The majority (90.3%) reported being born in Canada and nearly 17% of participants identified as Indigenous. Whereas most women rated their current health as fair (38.7%) or good (36.6%), 10.3% rated it as poor. Women rated their mental health status much lower with 34.1% reporting that their mental health was poor, 42.3% fair, 16.9% good and 6.6% very good or excellent. Most women (80.7%) reported that they were not currently living with their abusive partner.

**Table 2:** Demographic characteristics of the sample by study arm (N = 331)

	Control N = 156	Intervention N = 175		p-value
Age, mean (SD)	34.11 (10.09)	36.77 (9.25)		0.007
Parenting Child[ren] under 18 years old	59 (37.8%)	86 (49.1%)		0.038
Education, n (%)				
Some secondary school	16 (10.3%)	18 (10.3%)		0.062
Graduated from secondary school	34 (21.8%)	18 (10.3%)		
Some college or university	45 (28.8%)	53 (30.0%)		
Graduated from college or university	60 (38.5%)	84 (48.0%)		
Employment status, n (%)				
Employed full-time	40 (25.6%)	48 (27.4%)		0.703
Employed part-time	39 (25.0%)	37 (21.1%)		
Unemployed	77 (49.4%)	90 (51.4%)		
Difficulty living on total income, n (%)				
Not at all difficult	2 (1.3%)	12 (6.9%)		0.070
Some what difficult	44 (28.2%)	46 (26.3%)		
Difficult	34 (21.8%)	47 (26.9%)		
Very difficult	42 (26.9%)	36 (20.6%)		
Extremely difficult or impossible	34 (21.8%)	34 (19.4%)		
Community size, n (%)				
Rural community (less than 1,000 residents)	5 (3.2%)	5 (2.9%)		0.901
Small town (1,000 to 29,999 residents)	12 (7.7%)	14 (18.-%)		
Medium sized city (30,000 to 99,999 residents)	46 (29.5%)	58 (33.1%)		
Large city (more than 100,000 residents)	93 (59.6%)	98 (56.0%)		
Identified as Indigenous	28 (17.9%)	28 (16.0%)		0.637
Born in Canada	140 (89.7%)	159 (90.9%)		0.732
Health status				
Poor	12 (7.7%)	22 (12.6%)		0.462
Fair	63 (40.4%)	65 (37/1%)		
Good	60 (38.5%)	61 (34.9%)		
Very good or excellent	21 (13.4%)	27 (15.4%)		
Mental health status				
Poor	51 (32.7%)	62 (35.4%)		0.834
Fair	71 (45.5%)	69 (39.4%)		
Good	25 (16.0%)	31 (17.7%)		
Very good or excellent	9 (5.7%)	13 (7.5%)		

The vast majority (96.1%) reported that their abusive partner was a man, with a small group of 13 participants reporting the gender identity of their partner as: Trans woman, Trans man, genderqueer, or 2-spirited, with one response missing.

### Intervention delivery and safety

Of the 135 women who received some intervention, most (54.8%, n = 74) had between 10 and 18 visits with the nurse, while 37.0% (n = 50) had fewer and 8.1% (n = 11) had more. The average number of visits was 11.1 (SD = 6.1, Range 1–49). One woman who received an extremely high number of visits (i.e., 48) was dealing with a high risk situation with no other supports in place. The mean duration of the intervention was 5.8 months (SD = 1.59, range = 3 weeks – 8.9 months), with most (81.5%, n = 110) women engaged in iHEAL for between 5 and 7 months. Variation in the number of visits and intervention length were expected given that iHEAL is woman-led and tailored and because the complexity of issues women were addressing, and their resources, varied. Ratings collected from 127 women in the post-intervention survey supported high levels of acceptability and safety of the intervention. For example, while 46.4% of these women (n = 49) agreed that taking part in iHEAL was sometimes upsetting, 94.5% (n = 120) agreed or strongly agreed that they felt comfortable and safe in iHEAL visits, 5 women neither agreed or disagreed, and no women disagreed.

### Primary outcomes

As shown in Table 3, in the ITT analysis the intervention group had significantly greater short-term (baseline to 6 months) improvement in Quality of Life than the control group (p < .001, ES = 0.30), with this effect sustained longer-term (18 months) (p = .020, ES = 0.29). In the PP analysis, the effect sizes for both 6 and 18 months were stronger (ES = 0.35, 0.31, respectively) than in the ITT analysis.

**Table 3:** Means (SD) across time by group with significance of differences and Cohen’s d effect sizes at 6 and 18 months

	Control				Intervention				ITT^a^		PP^b^	
	Baseline (n = 156)	6 month (n = 127)	12 month (n = 121)	18 month (n = 127)	Baseline (n = 175)	6 month (n = 149)	12 month (n = 153)	18 month (n = 150)	Group x time p-values 6, 18 months	Effect Sizes 6, 18 months	Group x time p-values 6, 18 months	Effect Sizes 6, 18 months
**Primary Outcomes**												
Quality of Life	3.31 (1.07)	3.37 (1.15)	3.63 (1.27)	3.63 (1.29)	3.48 (1.26)	3.89 (1.32)	4.11 (1.34)	4.14 (1.39)	< 0.001* 0.020*	0.30* 0.29*	< 0.001* 0.009*	0.35* 0.31*
PTSD Symptoms	54.25 (13.69)	51.61 (14.53)	51.23 (15.87)	52.14 (15.11)	53.43 (14.74)	49.95 (16.89)	47.21 (16.88)	47.53 (17.38)	0.228 0.110	-0.06 -0.27	0.035* 0.018*	-0.19* -0.39*
**Secondary Outcomes**												
Depression	34.64 (13.44)	33.97 (14.37)	32.48 (15.69)	33.04 (14.93)	33.84 (15.06)	29.32 (15.70)	28.15 (15.79)	28.32 (16.55)	0.006* 0.063	-0.27 -0.27	< 0.001* 0.005*	-0.36* -0.37*
Confidence in Managing Daily Life	58.69 (18.83)	55.61 (20.32)	59.63 (20.40)	59.87 (23.03)	61.66 (19.61)	66.08 (22.01)	68.92 (19.41)	68.70 (20.60)	< 0.001* 0.006*	0.39* 0.30*	< 0.001* < 0.001*	0.56* 0.47*
Severity of IPV	20.38 (14.60)	12.67 (12.09)	13.24 (13.91)	13.67 (15.30)	20.81 (16.12)	11.02 (11.87)	10.63 (13.69)	7.81 (11.03)	0.172 0.006*	-0.13 -0.41*	0.046* 0.009*	-0.23* -0.45*
Chronic Pain	33.78 (26.63)	40.08 (26.90)	41.57 (25.71)	41.22 (27.76)	30.74 (26.43)	37.23 (30.14)	35.99 (30.01)	36.98 (31.95)	0.756 0.825	0.01 -0.05	0.447 0.930	-0.06 -0.05

Legend: *p < .05

^a^Intention-to-Treat Analysis

^b^Per Protocol Analysis with 135 who received at least 1 iHEAL visit

In the ITT analysis for PTSD Symptoms, the group by time interaction was not significant at either 6 (p = .228, -0.06) or 18 months (p = .110, ES = -0.27); however, in the PP analysis, the intervention group had a significantly greater improvement in PTSD Symptoms when compared to control for both 6 and 18 months (p = .035, ES = -0.19; p = .018, ES = -0.39). Thus, when delivered as planned, iHEAL was effective in improving women’s Quality of Life and PTSD Symptoms immediately post-intervention, with these changes maintained 1 year post-intervention.

### Secondary outcomes

For Confidence in Managing Daily Life, there were significant group by time effects favouring the intervention group in the ITT analysis, immediately post intervention (p = < 0.001, ES = 0.39) and 1 year post-intervention (p = .006, ES = 0.30). In the PP analysis, effect sizes were stronger at both time points (ES = 0.56, 0.47 at 6, 18 months, respectively).

The intervention group also had a greater initial reduction in Depressive Symptoms than the control group in both ITT and PP analysis (p = .006, ES=-0.27; p < .001, ES=-0.36). At 18 months, these effects were not sustained in the ITT analysis (p = .063, ES=-0.27) but remained significant in the PP analysis (p = .005, ES =-0.37).

Severity of IPV declined over time in both intervention and control groups, with a significantly greater initial post-intervention reduction in the PP analysis (p = .046, ES=-0.23) but not in the ITT analysis (p = .009, ES= -0.13). However, at 18 months, the group by time effects were significant in both analyses in favour of the intervention group, with moderate effects observed (ES=-0.41 in ITT, ES=-0.45 in PP). Thus, when delivered as planned, iHEAL was more effective than the control in increasing women’s Confidence in Managing Daily Life, and reducing Depression and Severity of IPV immediately post-intervention, with changes sustained 1 year later.

Level of Chronic Pain was relatively stable across time in both groups, although trending toward slightly increased disability. However, neither short- or longer-term group effects related to changes in chronic pain were observed in any analysis.

### Patterns of change in outcomes

Looking across primary and secondary outcomes in the PP analysis, three distinct patterns of effects are evident when comparing immediate (post-intervention) effects and longitudinal (1 year post intervention) effects (Table 4). First, for most outcomes (i.e., Quality of Life, Depression, Confidence in Managing Daily Life), consistent moderate effects were seen immediately post-intervention and those effects were sustained 1 year later. However, for PTSD Symptoms and Severity of IPV, there were weaker initial effects that grew stronger over time, suggesting a delayed effect. Finally, there were no effects on pain disability.

**Table 4:** Summary of Longitudinal Patterns of Change in Outcomes

Pattern of Change	Associated Outcomes
Moderate Initial Positive Effects, Sustained Over Time	Quality of Life Confidence in Managing Daily Life Depression
Weaker Initial Positive Effects, Strengthened Over Time	PTSD Symptoms IPV Severity
No Change	Chronic Pain

## Discussion

Results of this study provide the first evidence of the effectiveness of the Intervention for Health Enhancement and Living (iHEAL), a health promotion intervention that has been shown to be both acceptable and safe for women in the transition of separating from an abusive partner. Specifially, when delivered as planned, compared to usual care, iHEAL demonstrated positive effects on women’s Quality of Life and PTSD symptoms (primary outcomes), Depression, Confidence in Managing Daily Life and IPV Severity (secondary outcomes) that were sustained 1 year post intervention; there was no effect on Chronic Pain. These results extend the evidence base for iHEAL from support for pre-post changes up to 6 months post-intevention in single group studies with no control group [34, 36] to demonstrating the sustained effectiveness of iHEAL against a usual care control on multiple outcomes over a substantial period of time.

The effects of iHEAL on both PTSD Symptoms and IPV Severity were relatively weak initially, yet increased to moderate levels by 18 months, suggesting that more time may be needed to capture the intervention’s impacts on these outcomes. In the context of limited evidence supporting the benefits of IPV interventions on PTSD symptoms [24], and interventions delivered by health care providers on women’s safety [24, 27], these findings suggest that longer-term assessment of these important outcomes may be needed in future trials to detect intervention effects if they exist.

Collectively, these results suggest that iHEAL, an intervention that is woman-led, concurrently addresses women’s health, well-being and safety, and is tailored to womens’ priorities and contexts, has important short- and longer-term benefits for women who are navigating the transition of separating from an abusive partner. Given that iHEAL was developed drawing on theory and research about women’s experiences of IPV and the transition of separation, whether iHEAL would benefit women who intend to stay with their abusive partners is not known. Effective interventions are needed for these women and for all people who experience IPV, including men and people who identify as non-binary. However, to be effective, these interventions need to grounded in research about their specific needs and contexts.

Women who have experienced IPV access health services at high rates [17] and often with complex needs, yet health care responses to IPV continue to be crisis-oriented and lag behind other services, in part, due to provider and system readiness [19]. Effective interventions for women experiencing IPV are limited, particularly those offered by health care providers, including Registered Nurses, yet are needed to improve women’s lives and alleviate the burden of IPV’s human and economic costs. As the largest group of health professsionals in Canada [56] and nearly 50% of the global health workforce [57], cultivating the capacity of Registered Nurses to provide more effective support to women who have experienced IPV has potential for significant, widespread impact. Adopting iHEAL as a model for the education and practice of nurses could substantially improve women’s safety, health and well-being if scaled up carefully and with fidelity. However, this requires further study through carefully designed implementation research.

As a complex intervention, we hypothesize that multiple mechanisms explain the benefits of iHEAL observed in this trial. Fundementally, the approach used in iHEAL is one that is oriented to strengths, hope, and building women’s capacities (knowledge, skills and resources) in a way that is woman-led and prioritizes her *choice* and *control*. The centrality of these iHEAL features has been supported in earlier feasibility studies of iHEAL [33–36]) and by a concurrent process evaluation of trial data, the results of which are forthcoming. This approach likely explains the significant and sustained impact of iHEAL on women’s Confidence in Managing Daily Life, a broadly concept that aligns with the six components of iHEAL, and one of the strongest effects observed in this trial. Regaining confidence, control and hope for the future have been proposed as important indicators of healing from the impact of violence and/or trauma [58]; iHEAL is oriented to helping women in the transition of separation to persist in working on their priorities in spite of considerable obstacles. While additional analyses are needed to more fully understand mechanisms of effect, increased confidence across multiple domains is an important initial effect of iHEAL that could provide a foundation for improvements in other oucomes, including depression and QOL (i.e. satisfaction with multiple life domains).

Although rarely used as an outcome in IPV intervention research, QOL is an ideal outcome for this trial. Given the persistent health, social and economic conequences of IPV for many women, Jaradat and colleagues [48] argue that, when broadly conceptualized, “QOL can be considered an indicator of both the toll of IPV on women’s lives and their healing from the effects of abuse” (p. 2). In the context of this trial, QOL fits with the comprehensive nature of iHEAL and the breadth of it’s theorized impacts. Results of this trial extend a small body of research on QOL among women who have experienced IPV, including Sullivan and Bybee’s [46] classic RCT of post-shelter advocacy for women who had experience IPV which demonstrated sustained impacts on women’s QOL and physical violence. These are also key outcomes of advocacy interventions identified in theoretical work [59] and systematic reviews [26].

The long-term benefits of iHEAL in reducing IPV severity is important since few IPV interventions delivered by health care providers have been shown to reduce violence. iHEAL and advocacy interventions share some common elements, particularly related to safety planning and system navigation. For example, iHEAL nurses explored safety issues with women, conducted risk assessments, discussed safety strategies, helped women connect with specialized services and navigate a wide range of systems if they wished. This is a more robust approach to safety in comparison to usual health care practice which tends to emphasize identification of IPV and referral [21]. Extending the expertise of heath care providers to help women develop their own capacity to identify and address safety issues – particularly when done in collaboration with violence-specific services - could realize benefits not seen in other studies.

There is growing evidence that women’s mental health improves over time after separation from an abusive partner [6, 60] and that many types of IPV interventions have short-term benefits for depression but not PTSD symptoms [27]. Results of this trial add new evidence supporting the sustained benefits of iHEAL for *both* women’s depression and PTSD symptoms. As a health promotion intervention, iHEAL nurses assist women to identify and manage distressing symptoms, including both depression and trauma symptoms and, when needed, to navigate complex systems and access other services. It is likely that the benefits of iHEAL for women’s health are linked to each of these activities, and possibly to improvements in other areas of women’s lives, such as improved confidence and a reduction in IPV. In the post-separation context, evidence from longitudinal studies consistently shows less improvement in mental health in the context of ongoing IPV [6].

While all but two women completed the intervention prior to the onset of the COVID-19 pandemic in March 2020, the majority of post-intervention surveys were conducted when significant physical distancing restrictions were in place in Canada. There is substantial evidence of increased mental health concerns in response to social isolation and stress during this period in the population at large, inclusive of women experiencing IPV [61]. In this context of increased stress and uncertainly, that iHEAL showed moderate effects in improving mental health is noteworthy. In addition to supporting women to address the effects of violence, it is possible that iHEAL visits provided a buffer against the deleterious effects of COVID-19 stress, although the extent to which this is the case is uncertain.

The impacts of iHEAL on PTSD symptoms were initially more modest than for depession, but grew stronger over time. As a complex trauma, the traumatic effects of IPV may be more difficult to shift in the short-term [29], particularly when women have also experienced other forms of violence or trauma in their lifetimes [5, 62]. Concurrent chronic stressors (i.e., economic challenges, or experiences of stigma or discrimination when seeking help) can reinforce and activate trauma responses. Although not conceptualized as such, some of the goals of iHEAL are similar to what Herman describes as the ‘stabilization’ phases of healing from complex trauma, where the focus in not on therapy or changing the meaning of the trauma directly, but helping the woman ensure that basic needs for safety, health and support are met. This points to the importance of trauma- and violence-informed care as a universal approach in health care. Further, attention was given to engaging women in ways that prioritized their emotional safety (as well as physical safety); for example, clear expectations that telling her trauma story was not a requirement for support allowed women to be in control of their own narratives. For many women, especially those living in rural areas or on low incomes, access to specialized PTSD and trauma services is limited by lack of services, lengthy waitlists and costs; iHEAL shows promise in reducing PTSD symptomology suggesting its potential as a more accessible option for women who have experienced IPV.

As with our prior studies [34, 36], there was no improvement in chronic pain, pointing both to the refractory nature of chronic pain and the lack of effective supports in Canada for people living with pain [63]. Training for iHEAL nurses on chronic pain specifically drew attention to chronic pain as the embodiment of trauma. However, the medically dominated approach to the management of chronic pain, the fact the women’s pain is often dismissed, and the limited supports available [64], left both nurses and women with few options.

### Limitations

The broad community-based recruitment strategy employed in this study resulted in a sample that was relatively diverse, but with the requirement of English proficiency, there was limited inclusion of newcomers and women whose language of origin was not English. Representation by LBTQ + women was also limited despite intentional recruitment efforts. While iHEAL was designed for inclusivity, additional research is needed to more fully explore its effectiveness with specific groups of women, including whether those who face the most significant social and economic challenges benefit, and to explain the mechanisms by which iHEAL produces effects for varied outcomes. These and other analyses are in process. Further, the use of online advertisements and flyers to recruit the study sample was effective and these strategies are showing promise in early implementation research focussed on offering iHEAL as a program within various community-based health organizations. Understanding how to optimize the reach of the program remains a critical issue for implementation and scale up.

Although randomization produced good balance across the intervention and control group overall, those in the intervention arm were slightly older (with the mean for both groups in the mid-30’s) and more likely to be parenting dependent children. There is a substantial body of research documenting the challenges of mothering in the context of IPV, including surveillance and judgments from services and from the public, and ongoing violence and coercive control [8]. In this context, it is unlikely that mothering produced advantages for these women that could have systematically improved trial outcomes for them.

Retention rates in this trial were high (i.e.,84% of women at 18 months) and, since all cases were included in the analysis (using GEE), it is unlikely that attrition had a significant effect on the results. Despite high retention rates, 40 women randomized to the intervention arm did not participate in any nurse visits, largely due to instability in their lives and systemic barriers. Given the complexity of women’s lives during the transition of separation, it is noteworthy that so *few* women were *unable* to engage in the intervention. While the per protocol analysis helped mitigate the effects of non-engagement in testing the effects of the intervention on study outcomes, the challenges of providing support to women who have experienced or are experiencing IPV remain. In spite of the flexible, woman-led model, all women may not have seen iHEAL as right for them. Women who did not engage in the intervention were more likely to be receiving social assistance than those who engaged, suggesting that economic barriers or other stressors associated with living on lower income might have been a barrier to participation. In the context of this trial, the iHEAL nurses were able to engage women using a high level of flexibility and outreach by accommodating the women’s schedules and preferred meeting places, providing assistance with child care and transportation costs, and responding to “no shows” without judgement. This may pose a challenge for future integration of iHEAL within existing health services unless these critical supports and flexibility can be maintained.

## Conclusions

Women experiencing IPV face a wide range of challenges in the transition of separation from an abusive partner that often persist over time. Atlhough women have been shown to seek help from many types of services, including health care, few interventions address the breadth and complexity of women’s needs and priorities or have been shown to produce multiple benefits. The result of this study demonstrate that iHEAL, a health promotion intervention that offers broad, woman-led, tailored support across a range of issues, has initial and longer-term benefits for women’s quality of life, health, well-being and safety that are sustained over time, and provides novel evidence about the role of specially trained Registered Nurses in offering effective support to women. These promising results provide a strong foundation for broader implementation and scale up of iHEAL in Canada, with potential to adapt and test this effective intervention in other countries.

## Data Availability

The datasets generated and/or analyzed for this study are not publicly available due to issues of safety, sensitivity and privacy. Participants in this study consented only to sharing of their research data within the immediate team and not with external parties. Inquiries about access to data should be directed to the Principal Investigator (Marilyn Ford-Gilboe).

## Abbreviations

CASR-SF: Composite Abuse Scale (Revised)-Short Form
CESD-R: Center for Epidemiologic Studies Depression Scale, Revised
DSMC: Data Safety Monitoring Committee
GEE: Generalized Estimating Equations
iHEAL: Intervention for Health Enhancement and Living
IPV: Intimate Partner Violence
ITT: Intent-to-Treat
LBTQ+: Lesbian, bisexual, transgender, queer, and other identities
PCL-C: PTSD Checklist Civilian Version
PTSD: Post-Truamatic Stress Disorder
PP: Per Protocol
QOL: Quality of Life Scale
RCT: randomized control trial
SES: socioeconomic status
TVIC: Trauma- and Violence-Informed Care
VAS: visual analogue scale
WHO: World Health Organization
